# Tuning Intramolecular Charge Transfer in Antimony(V) Porphyrin through Axial Fluorination

**DOI:** 10.1021/acsomega.4c01773

**Published:** 2024-05-13

**Authors:** Noah Holzer, Jatan K. Sharma, Francis D’Souza, Prashanth K. Poddutoori

**Affiliations:** † Advanced Materials Center, University of Minnesota Duluth, 1405 University Drive, Duluth, Minnesota 55812, United States; ‡ Department of Chemistry & Biochemistry, University of Minnesota Duluth, 1038 University Drive, Duluth, Minnesota 55812, United States; § Department of Chemistry, University of North Texas, 1155 Union Circle, # 305070, Denton, Texas 76203-5017, United States

## Abstract

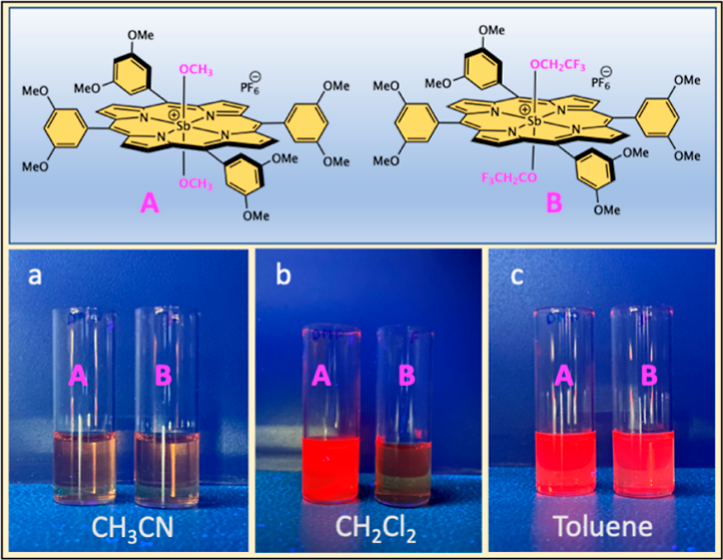

Modulation of intramolecular charge transfer (ICT) has been tested in two antimony(V) porphyrins, SbT(DMP)P(OMe)_2_·PF_6_ and SbT(DMP)P(OTFE)_2_·PF_6_, where the *meso*-positions are occupied by 3,5-dimethoxyphenyl (DMP), and the axial positions are linked with either methoxy (OMe) or trifluoroethoxy (OTFE) units, respectively. The presence of the Sb(+5) ion makes the porphyrin center electron poor. Under this situation, placing electron-rich units in the *meso*-position creates a condition for push–pull type ICT in the SbT(DMP)P(OMe)_2_·PF_6_. Remarkably, it is shown that the ICT character can be further enhanced in SbT(DMP)P(OTFE)_2_·PF_6_ with the help of electron-withdrawing TFE units in the axial position, which makes the porphyrin center even more electron scarce. The steady-state and transient studies as well as solvatochromism studies establish the ICT in SbT(DMP)P(OMe)_2_·PF_6_ and SbT(DMP)P(OTFE)_2_·PF_6_, and the strength of the ICT can be modulated by exploiting the structural properties of antimony(V) porphyrin. The existence of ICT is further supported by density functional theory calculations. The transient studies show that upon excitation of these porphyrin, their charge-transfer states convert to a full charger-separated states with appreciable lifetimes.

## Introduction

Intramolecular charge transfer (ICT) is the transfer of charge from an electron-rich part of the molecule to an electron-poor part of the same molecule.^1^ Recently, there has been a lot of interest in such molecules because of their applications in solar energy conversion,^2−15^ nonlinear optical materials,^16−18^ and organic-light-emitting diodes.^19^ Among the reported donor–acceptor (D–A) systems, porphyrin molecules emerged as one of the suitable compounds because they are photoactive and absorb light in the visible region; moreover, their photophysical and redox properties can be easily tuned by functionalization at the peripheral positions.^20^ Introducing electron-rich and electron-poor units on opposite sides of porphyrins have presented fascinating push–pull type molecules with unidirectional electron flow from the donor side to the acceptor side of the molecule.^13,21−26^ This induced directionality is ideal for use in solar energy capture and conversion applications.

Much of the interest in using porphyrin molecules for designing charge-transfer systems originates from the ease of controlling and customizing the optical and redox properties using their structural capabilities. Among porphyrins, main-group porphyrins make up a special class of molecules where the structural and redox properties can also be tuned by the central element. For example, the insertion of antimony(+5) and phosphorus(+5) ions in the porphyrin cavity results in an electron-deficient porphyrin center with two functionally active axial bonds.^27−30^ Recently, our group has employed the above criteria to synthesize a series of antimony(V) porphyrins and phosphorus(V) porphyrins, where the *meso*-positions were decorated with different degrees of methoxy phenyl substitutions.^27,28^ The presence of electron-rich methoxyphenyl groups induced ICT in the molecules from the peripheral methoxyphenyl units to the central antimony(V) or phosphorus(V) porphyrin ring. This ICT character was determined to result from the large electrostatic potential difference between the core of the porphyrin and its peripheral substitutions. Moreover, the study demonstrated that by changing the number and position of electron-donating groups on the *meso*-positions of the porphyrins, the resulting electrostatic potential difference between the core and periphery of the molecules could be altered. This provides a method by which the strength of the ICT character can be feasibly tuned.

**Scheme 1:**
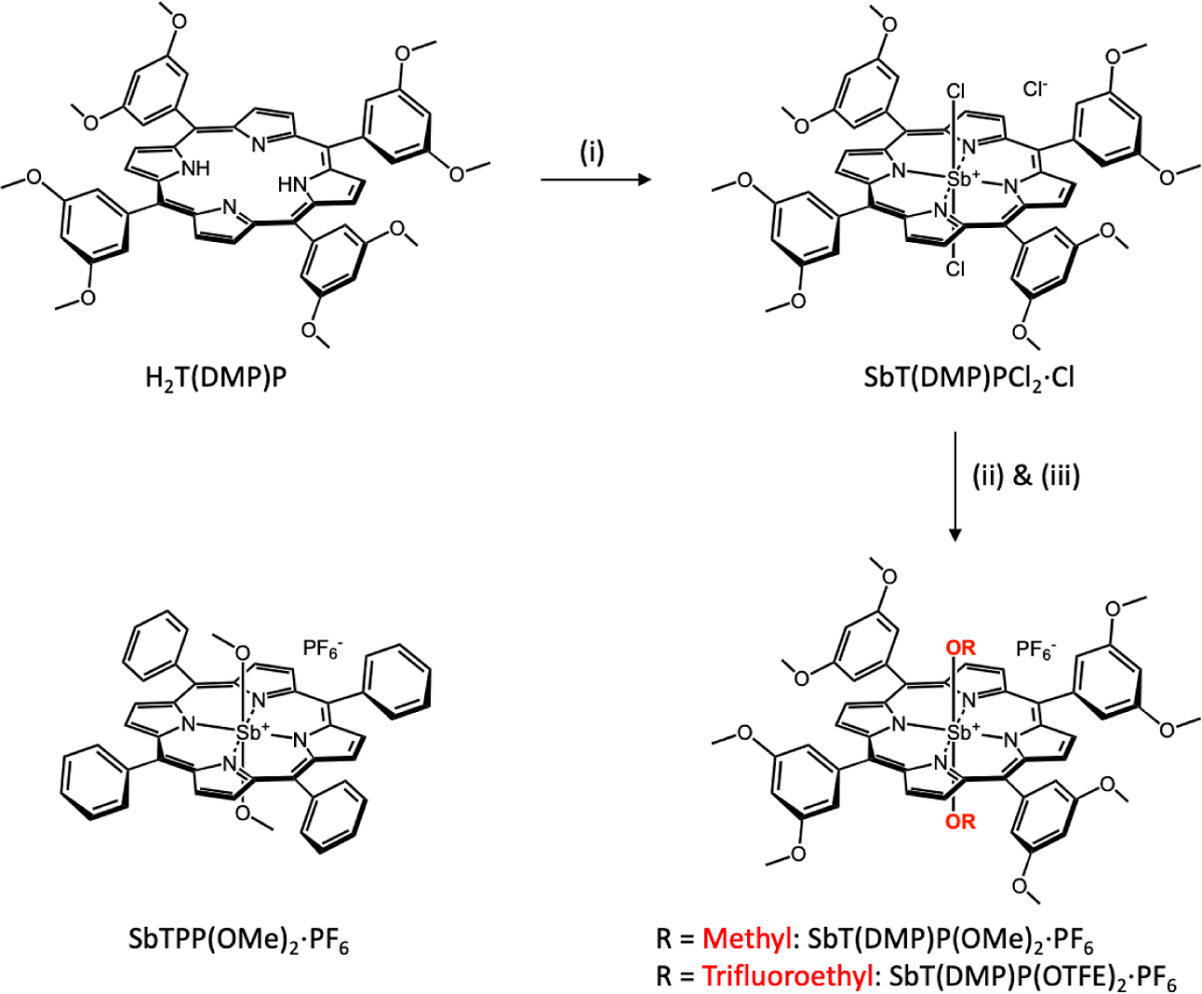
Investigated Antimony(V) Porphyrins and Their Synthesis. Reaction Conditions: (i) SbCl_5_, Pyridine (ii) CH_3_OH or CF_3_CH_2_OH, CHCl_3_, Pyridine, and (iii) NH_4_PF_6_

Alternatively, it was speculated that by keeping the electron density fixed at the peripherals (or *meso*-phenyl rings) and increasing electron-deficiency at the core of the antimony(V) porphyrin, the strength of the ICT character could also be much improved. To test this hypothesis, two antimony(V) porphyrins, SbT(DMP)P(OMe)_2_·PF_6_ and SbT(DMP)P(OTFE)_2_·PF_6_, were prepared and investigated in the present study, as seen in Scheme 1. From our preceding studies, the ICT was shown to exist from the peripheral dimethoxyphenyl unit to the central antimony(V) ion in SbT(DMP)P(OMe)_2_·PF_6_.^28^ From solvatochromism studies, it was found that the observed ICT is moderately strong as it manifested in only very polar solvents, such as CH_3_CN. To test the above hypothesis and to increase the ICT character without changing the peripheral substitutions in SbT(DMP)P(OMe)_2_·PF_6_, a new compound, SbT(DMP)P(OTFE)_2_·PF_6_, has been prepared, in which the electron deficiency at the center was increased by axial functionalization using trifluoroethoxy (TFE) units. As predicted, the optical and computational data show that the SbT(DMP)P(OTFE)_2_·PF_6_ exhibits relatively strong ICT, compared to SbT(DMP)P(OMe)_2_·PF_6_, as it exhibits the ICT even in the moderate polar solvent CH_2_Cl_2_. The study establishes the importance of different modes of functionalization (*meso*- and axial-) to tune the ICT properties of the investigated molecules. Furthermore, the study also indicates that it is possible to tune the ICT character by either altering the electron density of the peripheral substitutions or the electron deficiency of the central Sb(+5) ion, resulting in a larger electrostatic potential difference within the structure of the molecule. It is important to note that the studied ICT was induced without complex structural modifications on the porphyrin molecule. Additionally, this study highlights the importance of the Sb(+5) ion in the porphyrin cavity, without this ion the ICT is simply not achievable in these systems.^31^ The combination of these properties makes the studied systems potential candidates for artificial photosynthesis; providing directionality to electron flow to improve solar energy conversion and storage efficiencies.

## Experimental Section

### Materials

The chemicals and solvents utilized in this study were purchased from Sigma-Aldrich, Tokyo Chemical Industry (TCI), Accela, Alfa-Asear, Fisher Chemical, and Acros Organics and were used as received. Anhydrous solvents were used in all of the performed reactions. Chromatographic materials were purchased from SiliCycle or Sigma-Aldrich. Synthesis of the H_2_T(DMP)P, SbT(DMP)PCl_2_·Cl, SbTPP(OMe)_2_·PF_6_, and SbT(DMP)P(OMe)_2_·PF_6_ were reported elsewhere along with the SbTPP(OMe)_2_·PF_6_ used a reference in spectroscopic and analytical methods.^28^ The synthesis of SbT(DMP)P(OTFE)_2_·PF_6_ is described in the below section.

### Synthesis of SbT(DMP)P(OTFE)_2_·PF_6_

SbT(DMP)PPCl_2_·Cl (29.4 mg, 0.21 mmol) was dissolved in a mixture of 1 mL of anhydrous pyridine, 2 mL of 2,2,2-trifluoroethanol, and 2 mL of chloroform (amylene stabilized; no ethanol). The resulting solution was stirred at 80 °C under an inert atmosphere for 5 h. The solvent was removed under reduced pressure to obtain the crude product. To make the purification easier counterion exchange, i.e., Cl^–^ to PF_6_^–^, was performed by redissolving the crude product in 10 mL of CH_3_CN. To this 150 mg of NH_4_PF_6_ was added followed by 50 mL of water to induce precipitation. The precipitate was collected via filtration and purified by using silica gel column chromatography. The product was dry packed onto the silica, and the column was eluted first with CH_2_Cl_2_ to remove low polar bands and then with CH_2_Cl_2_/ethyl acetate (=96:4) to elute the product. At this stage, some portion of the product contains 2,2,2-trifluoroethoxide as a counterion. To protonate the 2,2,2-trifluoroethoxide and replace the counterion with Cl^–^, the product was stirred in 2 mL of CH_3_CN and 1 mL of HCl (1 M) for 5 min. The solution was extracted with CH_2_Cl_2_, and the organic layer was water washed, and then the solvent was evaporated. Finally, the counterion exchange, i.e., Cl^–^ to PF_6_^–^, was repeated to obtain the pure product as a violet solid. Yield: 22 mg (62%). ESI-MS: *m*/*z* 1171.2409 for [M – PF_6_]^+^, calcd, 1171.2307 for C_56_H_48_F_6_N_4_O_2_Sb^+^. ^1^H NMR (CDCl_3_, 400 MHz): δ, ppm 9.67 (s, 8H), 7.60 (s, 8H), 7.03 (s, 4H), 4.02 (s, 24H), −1.84 (q, 4H, *J* = 8.7 Hz). 19F NMR (CDCl3, 375 MHz): δ, ppm −73.8 (d, 6F, *J* = 712.0 Hz), −78.9 (s, 6F).

## Results

### Synthesis and Characterization

The reactions employed to obtain the target molecules are summarized in Scheme 1. Free-base porphyrin H_2_T(DMP)P, and its antimony(V) derivatives, SbT(DMP)PCl_2_·Cl, and SbT(DMP)P(OMe)_2_·PF_6_, were prepared by the earlier published methods.^28^ The axial Sb–Cl bonds of SbT(DMP)PCl_2_·Cl were reacted with trifluoroethanol to yield the [SbT(DMP)P(OTFE)_2_]^+^. At this stage, it was found that the [SbT(DMP)P(OTFE)_2_]^+^ exists as a mixture of two salts one with PF_6_^–^ and another with CF_3_CH_2_O^–^ counterions. The mixture was treated with 1 M HCl followed by counterion exchange to obtain target SbT(DMP)P(OTFE)_2_·PF_6_. The structure of SbT(DMP)P(OTFE)_2_·PF_6_ was established using the ESI-MS and NMR (^1^H, ^19^F, ^31^P) spectroscopy, as seen in Figure S1. The mass spectrum of SbT(DMP)P(OTFE)_2_·PF_6_ revealed an intense peak, which corresponds to the mass (*m*/*z*) of [M – PF_6_]^+^. The ^1^H NMR spectrum manifests a strong upfield chemical shift (−1.84 ppm) for the protons on the axial trifluoroethoxy unit due to the ring current effect of the porphyrin macrocycle. The ^19^F NMR spectrum revealed two different types of fluorine atoms (i.e., PF_6_^–^ and TFE units) on the porphyrin structure.

### Computational Studies

**Figure 1:**
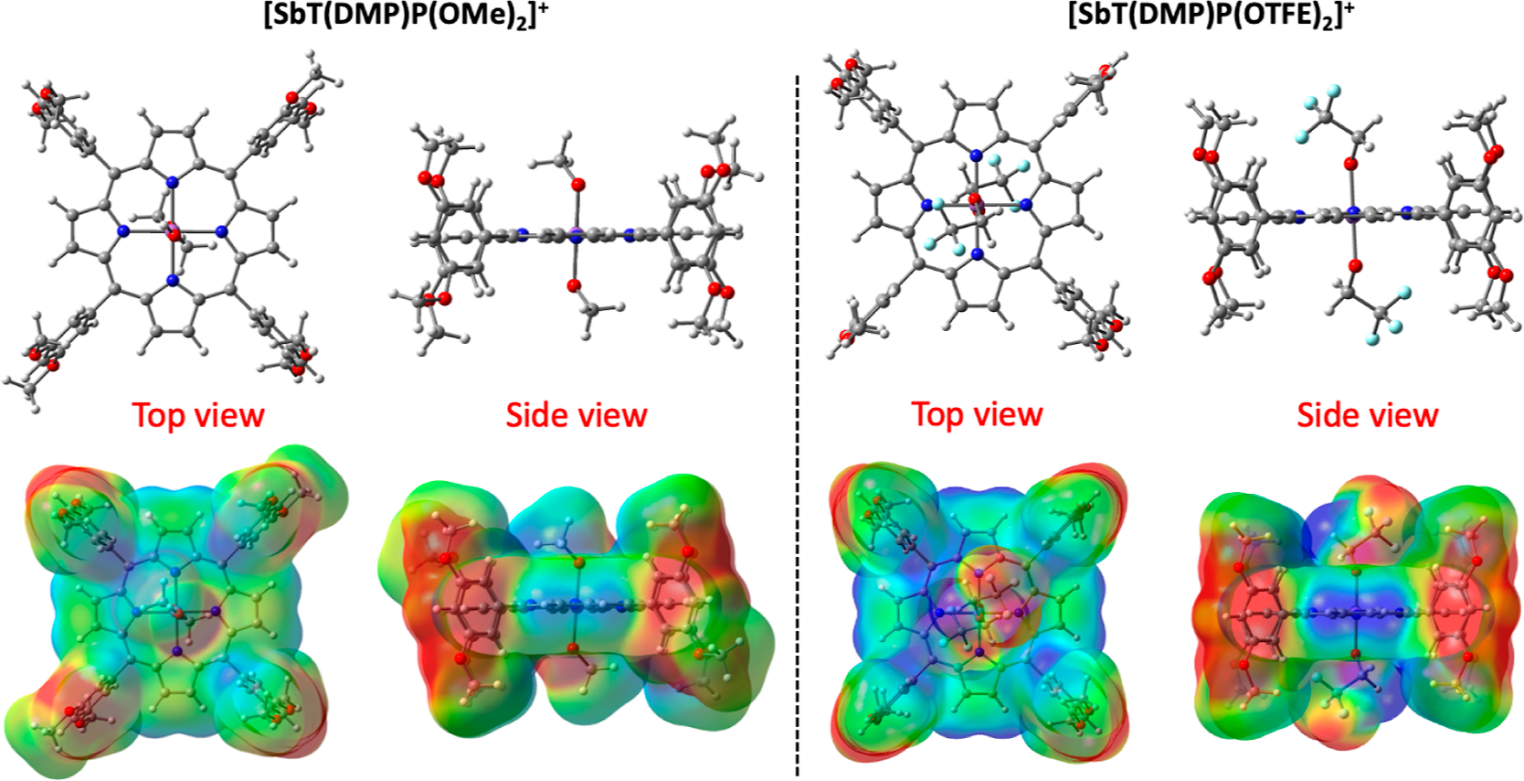
Top row shows the optimized structures and the bottom rows depict the electrostatic maps of [SbT(DMP)P(OMe)_2_]^+^ and [SbT(DMP)P(OTFE)_2_]^+^. The blue color indicates the electron acceptor region, and the red color indicates the electron donor region.

**Figure 2:**
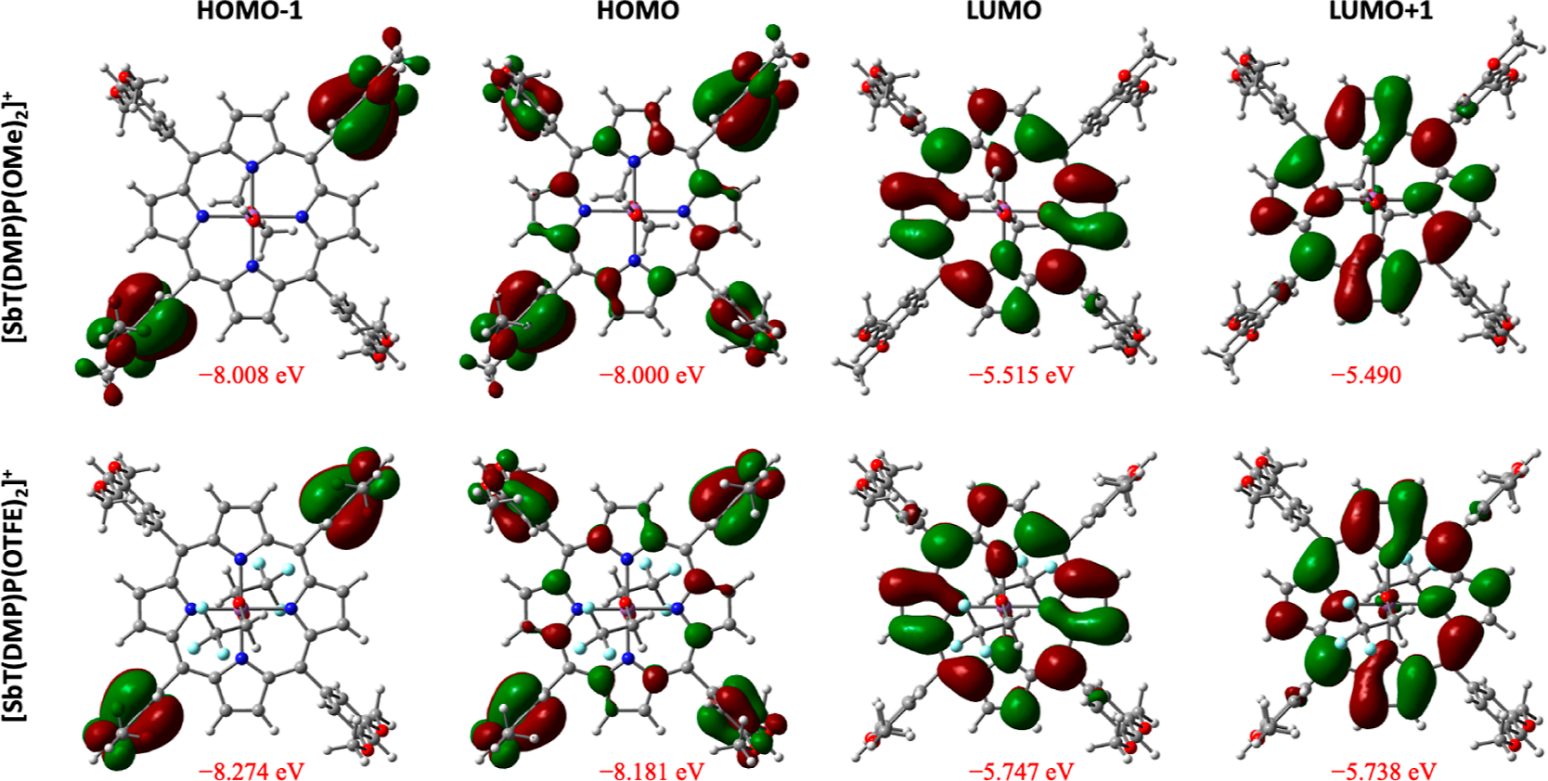
Computed molecular orbitals and corresponding energies of [SbT(DMP)P(OMe)_2_]^+^ and [SbT(DMP)P(OTFE)_2_]^+^.

The density functional theory (DFT) calculations were performed to rationalize the electronic structure differences in the investigated antimony(V) porphyrins. Figure 1 top row shows the optimized structures, and the bottom row reveals the electrostatic potential (ESP) maps. The optimized structures are consistent with the earlier reported crystal structures where the central porphyrin exists in planarity.^28,29^Figure 2 illustrates the frontier orbitals of [SbT(DMP)P(OMe)_2_]^+^ and [SbT(DMP)P(OTFE)_2_]^+^. The LUMO and LUMO + 1 are limited mainly to the porphyrin ring. However, HOMO and HOMO – 1 are largely delocalized over the porphyrin peripherals. A major component of the HOMO is predominantly on the *meso*-aryl ring with a minor component on the central porphyrin ring. Analogously, HOMO – 1 is found to be exclusively on the *meso*-aryl units. The presence of HOMO and LUMO on different parts within the molecule creates the conditions necessary for push–pull type ICT character.^28,29^ The calculated HOMO–LUMO gaps are 2.49 and 2.43 eV in [SbT(DMP)P(OMe)_2_]^+^ and [SbT(DMP)P(OTFE)_2_]^+^, respectively. It is noteworthy to mention that the computed HOMO–LUMO gap is in a vacuum, and no solvent matrix was incorporated in the calculation.

The blue and red regions in the ESP maps designate the electron acceptor and electron donor segments, respectively, in the molecule. This uneven electron distribution within the molecule is expected to induce the ICT character in both of the investigated compounds, [SbT(DMP)P(OMe)_2_]^+^ and [SbT(DMP)P(OTFE)_2_]^+^, from the peripheral DMP unit to the central porphyrin. Interestingly, careful investigation of the ESP maps reveals certain differences between [SbT(DMP)P(OMe)_2_]^+^ and [SbT(DMP)P(OTFE)_2_]^+^. The red color is roughly the same, but the blue color is certainly more pronounced in [SbT(DMP)P(OTFE)_2_]^+^. This is due to the presence of axial TFE electron-withdrawing units, which increases the electrostatic potential difference between the central ring and peripheral substitutions. Therefore, it is reasonable to expect that such disparity could result in a stronger ICT in [SbT(DMP)P(OTFE)_2_]^+^ compared to that of [SbT(DMP)P(OMe)_2_]^+^. However, it is important to mention that here the ICT prediction is purely based on computational studies. Therefore, supporting or establishing the ICT requires experimental evidence, consequently, a variety of spectroscopic and analytical methods were employed as discussed below.

### Steady-State Absorption Studies

**Figure 3:**
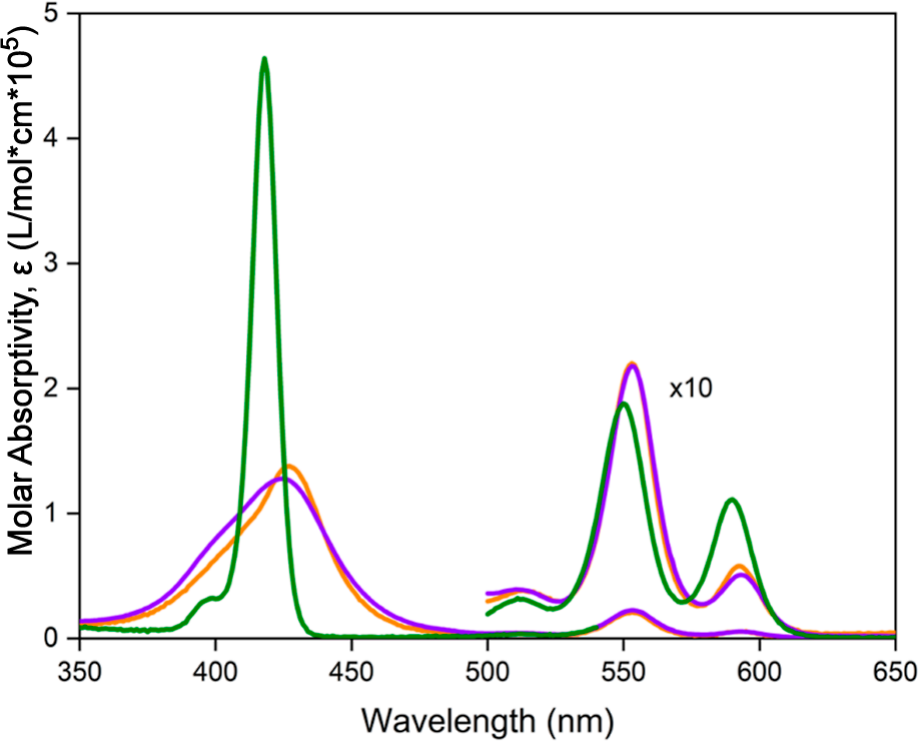
UV–visible absorption spectra of SbTPP(OMe)_2_·PF_6_ (green), SbT(DMP)P(OMe)_2_·PF_6_ (orange), and SbT(DMP)P(OTFE)_2_·PF_6_ (violet) in CH_3_CN.

The UV–visible absorption spectra of investigated porphyrins were measured in CH_3_CN, their corresponding spectra are shown in Figure 3 and the data is summarized in Table 1. For comparison, the absorption spectrum of SbTPP(OMe)_2_·PF_6_ (see Scheme 1 for structural information) is also depicted in Figure 3. Each antimony(V) porphyrin showed one high-energy transition (Soret band) and two low-energy transitions (Q-bands). The absorption spectra of SbT(DMP)P(OMe)_2_·PF_6_ and SbT(DMP)P(OTFE)_2_·PF_6_ are very similar to each other but significantly different from those of SbTPP(OMe)_2_·PF_6_. The presence of electron-rich DMP donor units, in SbT(DMP)P(OMe)_2_·PF_6_ and SbT(DMP)P(OTFE)_2_·PF_6_, results in significant perturbation of the electronic structure of porphyrin. Moreover, the degree of perturbation depends not only on the DMP donor ability but also on the acceptor ability of the central porphyrin ring. Careful observation reveals that the Soret of SbT(DMP)P(OTFE)_2_·PF_6_ is slightly broader than that of SbT(DMP)P(OMe)_2_·PF_6_ which could be due to a rise in the electrostatic potential difference between the peripheral and central porphyrin ring.

**Table 1:** Optical and Redox Data of the Investigated Antimony(V) Porphyrins in CH_3_CN

	absorption	potentials (V *vs* SCE)^a^	
sample	λ_max_ [nm] (log(ε[M^–1^ cm^–1^]))	reduction	oxidation
SbTPP(OMe)_2_·PF_6_	418 (6.01), 550 (4.61), 590 (4.40)	–0.33, −0.75	1.78
SbT(DMP)P(OMe)_2_·PF_6_	426 (5.79), 553 (4.97), 593 (4.39)	–0.32, −0.76	1.73
SbT(DMP)P(OTFE)_2·_PF_6_	425 (5.11), 553 (4.35), 593 (3.74)	–0.30, −0.70	1.74

a 0.1 M TBA·PF_6_ was used as a supporting electrolyte.

### Redox Chemistry

**Figure 4:**
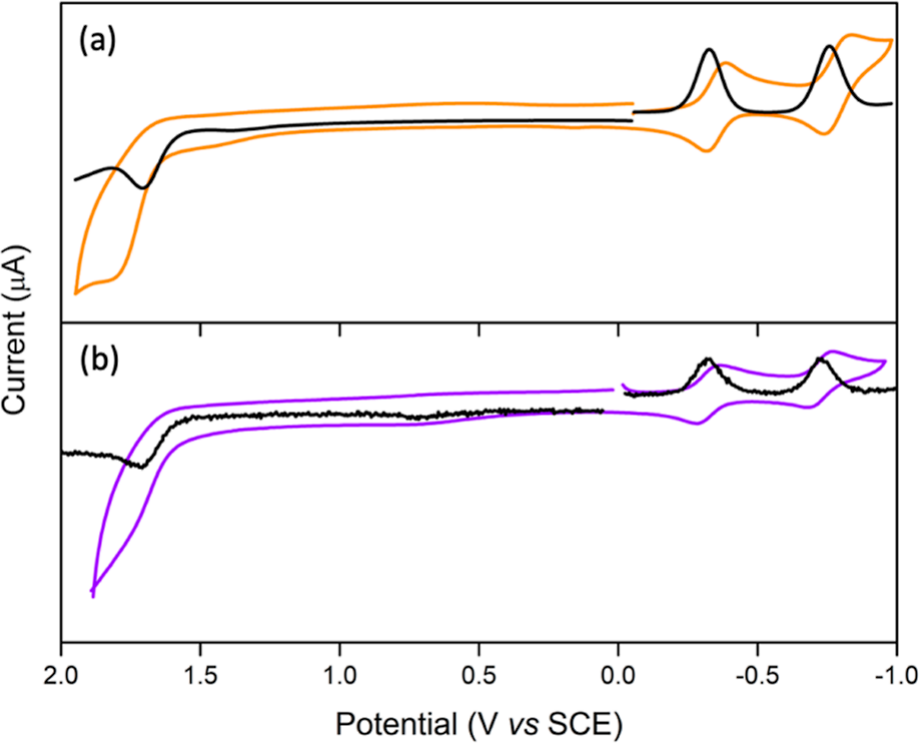
Cyclic (orange/violet) and differential pulse (black) voltammograms of (a) SbT(DMP)P(OMe)_2_·PF_6_ and (b) SbT(DMP)P(OTFE)_2_·PF_6_ in CH_3_CN with 0.1 M TBA·PF_6_.

Cyclic and differential voltammograms of SbT(DMP)P(OTFE)_2_·PF_6_ were measured in CH_3_CN with 0.1 M TBA·PF_6_. The collected voltammograms are shown in Figure 4 and the data are summarized in Table 1. For discussion, the electrochemistry data of SbTPP(OMe)_2_·PF_6_ and SbT(DMP)P(OMe)_2_·PF_6_ is adopted from the literature.^28^ The nature of the redox processes is established from the peak-to-peak separation values and the cathodic-to-anodic peak current ratios. Each of the investigated compounds displayed two reduction and one oxidation processes. The cathodic scan of SbT(DMP)P(OTFE)_2_·PF_6_ revealed two reduction processes at −0.30 and −0.70 V, whereas the anodic scan showed one oxidation process at 1.74. The reduction processes originate from the successive addition of two electrons to the LUMO, and the lone oxidation process originates from the removal of electrons from the HOMO. The nature of the reductions is found to be one-electron reversible processes. On the contrary, the oxidation process is found to be quasi-reversible. The HOMO of SbT(DMP)P(OTFE)_2_·PF_6_ is mainly localized on the *meso*-DMP; hence, the quasi-reversible nature arises from the oxidation of the DMP units.^31^ In general, the antimony(V) porphyrins hold very positive potentials within the hypervalent porphyrin family.^30^ The presence of Sb(+5) in a high oxidation state makes the porphyrin core highly electron deficient. Under this state, it is logical to expect high oxidation and low reductions for the studied systems. As predicted by the DFT calculations, the reduction potentials are slightly shifted toward positive potentials in SbT(DMP)P(OTFE)_2_·PF_6_ compared to SbTPP(OMe)_2_·PF_6_ and SbT(DMP)P(OMe)_2_·PF_6_ due to the electron-withdrawing nature of the TFE units in the axial positions.

### Energy Level Diagrams

**Figure 5:**
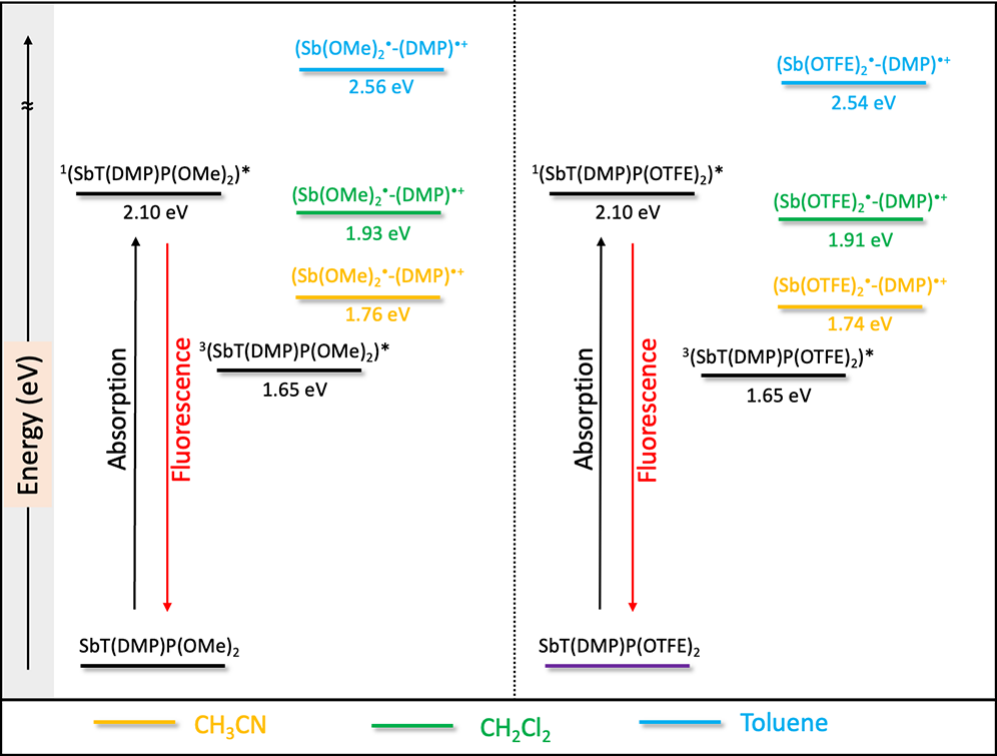
Estimated energy levels of SbT(DMP)P(OMe)_2_·PF_6_ and SbT(DMP)P(OTFE)_2_·PF_6_ in toluene, CH_2_Cl_2_, and CH_3_CN.

Figure 5 shows energy level diagrams of the SbT(DMP)P(OMe)_2_·PF_6_ and SbT(DMP)P(OTFE)_2_·PF_6_ compounds. The diagram was constructed by using the optical and redox data to study the possible charge-transfer processes. From the overlap of the absorption and fluorescence spectra in CH_3_CN (Figure S2), the excited singlet state energy (*E*_0–0_) was estimated to be ∼2.10 eV for the studied porphyrins. The triplet state energy (*E*_T_ = 1.65 eV) of SbT(DMP)P(OMe)_2_·PF_6_ is obtained from the literature.^28^ Based on the electronic structure, it is anticipated that the SbT(DMP)P(OTFE)_2_·PF_6_ will have a similar triplet state energy. The ICT state energy (*E*_CT_) was calculated using the following eqs 1 and 2.^32^
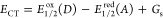
1
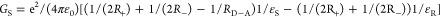
2

Here, *E*_1/2_^ox^ is the oxidation potential of free DMP unit (=1.50 V)^31^ and *E*_1/2_^red^ is the first reduction potential [−0.32 and −0.30 V for SbT(DMP)P(OMe)_2_·PF_6_ and SbT(DMP)P(OTFE)_2_·PF_6_, respectively] of the porphyrin. For this estimation, the oxidation potential of the free DMP was utilized because the DFT studies show that the HOMO is predominantly on the DMP unit. The *G*_S_ is the ion-pair stabilization and incorporates the solvent-dependent Coulomb energy change upon CT state formation. *R*_+_, *R*_–_, and *R*_D–A_ are the donor radius (2.74 Å), acceptor radius (4.30 Å), and center-to-center distance (6.34 Å) between donor and acceptor, respectively. These measurements were obtained from the crystal structure of SbT(DMP)P(OMe)_2_·PF_6_^31^ and it is anticipated that compound SbT(DMP)P(OTFE)_2_·PF_6_ will have similar parameters. ε_S_ is the dielectric constant of the solvent used for the photophysical studies (37.5, 8.93, and 2.38 for CH_3_CN, CH_2_Cl_2_, and toluene, respectively). ε_R_ is the dielectric constant of the solvent used for measuring the redox potentials, in this case CH_3_CN. Using the radii from the crystal structure parameters, *G*_S_ values of −0.06, 0.11, and 0.74 eV are obtained in CH_3_CN, CH_2_Cl_2_, and toluene, respectively. The calculated free-energy level diagram suggests that the charge transfer is energetically favorable in SbT(DMP)P(OMe)_2_·PF_6_ and SbT(DMP)P(OTFE)_2_·PF_6_ in polar CH_3_CN and moderately polar CH_2_Cl_2_, but not in the nonpolar toluene solvent. Such solvent polarity-dependent charge transfer is well established in the literature.^33,34^

### Solvatochromism

**Figure 6:**
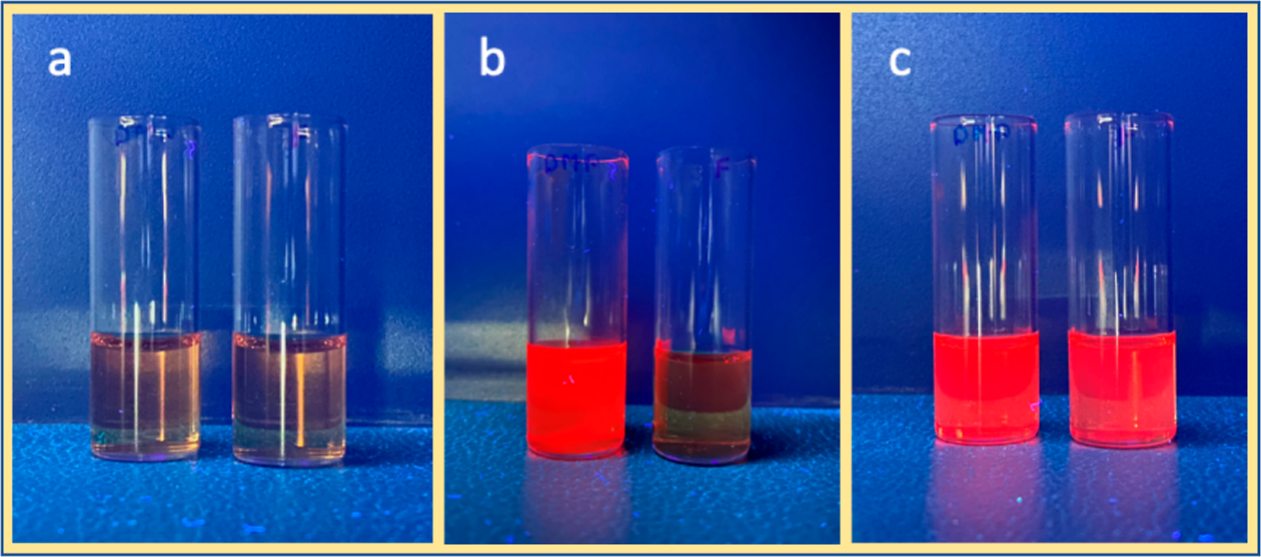
Solvatochromism of SbT(DMP)P(OMe)_2_·PF_6_ (left vial) and SbT(DMP)P(OTFE)_2_·PF_6_ (right vial) under 365 nm UV light in (a) CH_3_CN, (b) CH_2_Cl_2_, and (c) toluene.

To prove the ICT character, solvatochromism studies were performed in CH_3_CN, CH_2_Cl_2_, and toluene. Figure 6 shows the images of SbT(DMP)P(OMe)_2_·PF_6_ and SbT(DMP)P(OTFE)_2_·PF_6_ solutions taken under UV irradiation (365 nm). The solution concentrations were maintained at ∼1 × 10^–4^ M. The proposed ICT should generate the porphyrin molecule with partial charges and should be sensitive to the solvent polarity. As predicted, in polar CH_3_CN solutions, both compounds SbT(DMP)P(OMe)_2_·PF_6_ and SbT(DMP)P(OTFE)_2_·PF_6_ are nonemissive. In moderate polar CH_2_Cl_2_, fluorescence from SbT(DMP)P(OMe)_2_·PF_6_ is fully recovered, but not from SbT(DMP)P(OTFE)_2_·PF_6_. Interestingly, both compounds become fluorescent in nonpolar toluene solutions. The observed solvent-dependent emission property signifies the ICT character of these molecules. Moreover, this study differentiates these two porphyrins based on the ICT character; enhanced ICT is observed in SbT(DMP)P(OTFE)_2_·PF_6_ due to the presence of the fluoroalkyl substituents in the axial position.

### Steady-State and Time-Resolved Fluorescence Studies

**Figure 7:**
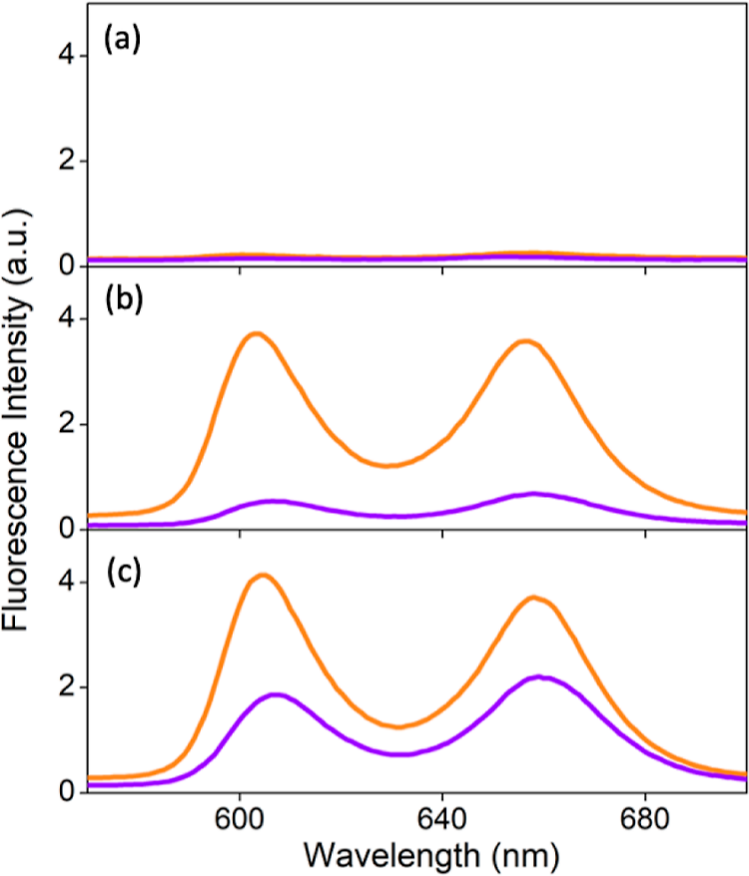
Steady-state fluorescence spectra of SbT(DMP)P(OMe)_2_·PF_6_ (orange) and SbT(DMP)P(OTFE)_2_·PF_6_ (violet) in (a) CH_3_CN, (b) CH_2_Cl_2_, and (c) toluene. Excitation wavelength: 550 nm.

The steady-state fluorescence spectra were measured in CH_3_CN, CH_2_Cl_2_, and toluene at an excitation wavelength of 550 nm with solutions containing equal optical densities. The spectra are shown in Figure 7 and the data are summarized in Table 2. As shown in Figure 7, the spectra are similar in shape, but the fluorescence quantum yield of SbT(DMP)P(OTFE)_2_·PF_6_ is notably smaller than that of SbT(DMP)P(OMe)_2_·PF_6_. The difference in quantum yields of these compounds is most pronounced in CH_2_Cl_2_, indicating that ICT is stronger in SbT(DMP)P(OTFE)_2_·PF_6_. This trend remains valid in nonpolar toluene solutions. The dramatic fluorescence quenching observed in CH_3_CN and CH_2_Cl_2_ suggests that the axial TFE groups can withdraw some electron density from the porphyrin π-system through the central antimony(V) ion, increasing the potential difference between the porphyrin macrocycle and the meso-aryl rings; resulting in the stronger ICT character in SbT(DMP)P(OTFE)_2_·PF_6_.

**Table 2:** Fluorescence Data of the Investigated Antimony(V) Porphyrins

sample	fluorescence λ_max_ [nm] (Φ_F_)^a^		
	average lifetime (τ, ns) (ChiSQ)^b^		
	CH_3_CN	CH_2_Cl_2_	toluene
SbTPP(OMe)_2_·PF_6_	598, 652 (0.040)	600, 654 (0.038)	601, 655 (0.045)
	1.36 (1.03)	1.24 (1.02)	1.04 (0.99)
SbT(DMP)P(OMe)_2_·PF_6_	600, 658 (0.003)	603, 656 (0.024)	605, 658 (0.026)
		1.02 (1.05)	0.91 (1.07)
SbT(DMP)P(OTFE)_2_·PF_6_	602, 654 (0.001)	607, 658 (0.006)	607, 659 (0.016)
		0.54(1.13)	0.73(1.02)

a Excitation wavelength = 550 nm.

b Excitation wavelength = 560 nm.

Time-resolved fluorescence studies of SbT(DMP)P(OTFE)_2_·PF_6_ were measured in CH_3_CN, CH_2_Cl_2_, and toluene. The time-resolved fluorescence data of the reference samples, SbTPP(OMe)_2_·PF_6_ and SbT(DMP)P(OMe)_2_·PF_6_, is obtained from literature.^28^ A 560 nm wavelength light was used to excite the samples, and the high-energy fluorescence band was used to collect the emission. Figure S3 depicts the fluorescence decay profiles and the corresponding lifetime data is summarized in Table 2. The average value was considered for discussion purposes. In CH_3_CN, both SbT(DMP)P(OMe)_2_·PF_6_ and SbT(DMP)P(OTFE)_2_·PF_6_ are found to be nonfluorescent, hence, their lifetimes could not be determined. As shown in Table 2, both SbT(DMP)P(OMe)_2_·PF_6_ and SbT(DMP)P(OTFE)_2_·PF_6_ revealed a decrease in lifetimes compared to SbTPP(OMe)_2_·PF_6_. The decrease in lifetimes with increasing solvent polarity complements the presence of charge-transfer characters in these molecules.

### Femtosecond Transient Absorption Studies

In order to spectrally prove the existence of excited state charge transfer as a function of the nature of the axial electron-rich/electron-deficient alkyl entity and their solvent polarity dependence, femtosecond transient absorption (*fs*-TA) studies were performed for SbT(DMP)P(OTFE)_2_·PF_6_ in polar CH_3_CN, moderately polar CH_2_Cl_2_, and nonpolar toluene. The results are compared with those of the earlier reported SbT(DMP)P(OMe)_2_·PF_6_, as summarized below. To help interpret the transient data, spectra of oxidized and reduced SbT(DMP)P(OTFE)_2_·PF_6_ were recorded in CH_3_CN, as shown in Figure S4. Such spectral characterization was reported earlier on SbT(DMP)P(OMe)_2_·PF_6_.^28^ Nitrosonium tetrafluoroborate as an oxidizing agent and cobaltocene as a reducing agent were utilized. During the oxidation process of SbT(DMP)P(OTFE)_2_·PF_6_, the diminished intensity of the neutral compound did not result in any new peaks, only a small increase in the 625–750 nm range was observed. This is understandable as the peripheral DMP groups are involved in oxidation and not in the porphyrin π-system. However, during the process of reduction, a broad peak spanning 625–850 nm with a peak maxima at 695 was observed. Generation of transient peaks in this spectral region upon photoexcitation of the SbT(DMP)P(OTFE)_2_·PF_6_ would provide direct proof for the occurrence of charge transfer.

The *fs*-TA of SbT(DMP)P(OMe)_2_·PF_6_ was recently reported by us and found that this compound revealed solvent-dependent charge transfer, that is, no charge transfer in nonpolar toluene (see Figure S5a) and charge transfer in polar CH_3_CN (see Figure S5b).^28^ For the T_1_ state, a lifetime of 11.8 μs in toluene was obtained from nanosecond transient spectral studies. On the contrary, in CH_3_CN, clear evidence of charge transfer was possible to secure that lasted for about 20.8 ps.^28^

**Figure 8:**
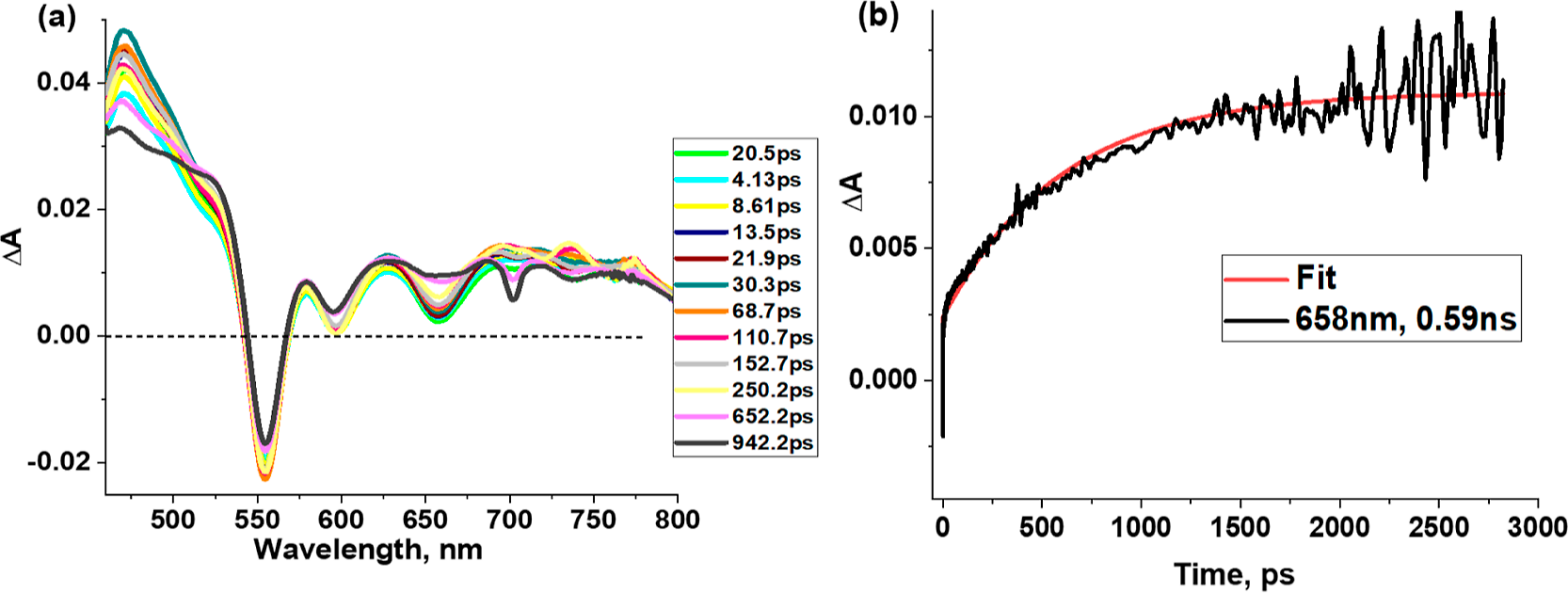
(a) *fs*-TA spectra at the indicated delay times of SbT(DMP)P(OTFE)_2_·PF_6_ in toluene with an excitation wavelength of 435 nm. (b) Decay profile of the 658 nm peak.

Figure 8a shows the *fs*-TA spectra at the indicated delay times for SbT(DMP)P(OTFE)_2_·PF_6_ in toluene at an excitation wavelength of 435 nm. The S_1_ state fully developed in about 20 ps from the initially formed S_2_ state revealed excited-state absorption bands at 470, 580, 628, 692, 719, and 760 nm. Negative peaks at 555, 596, and 658 nm were also observed. By comparison with the previously discussed absorption and fluorescence spectral data, the first two negative peaks at 555 and 596 nm to ground state bleaching (GSB) and the 596 and 658 nm peaks to stimulated emission (SE) were possible to assign. The decay of the ESA peaks and recovery of the GSB and SE peaks followed the same time profile and started developing new signals at 468, 522, and 685 nm due to triplet–triplet absorption of the triplet state formed by the process of intersystem crossing. The time profile of the 658 nm peak is shown in Figure 8b. A decay time constant of 0.59 ns, close to the previously discussed fluorescence lifetime, was observed. Expectedly, no evidence of a charge transfer state was observed. A sharp negative peak was observed at around 700 nm at higher delay times. Although the origin of this peak is not clear, this could be due to simulated emission of the triplet state; however, additional studies are needed to confirm this. The nanosecond transient spectra were also recorded; however, the signal-to-noise ratio was poor and a reliable lifetime for the triplet state could not be secured.

**Figure 9:**
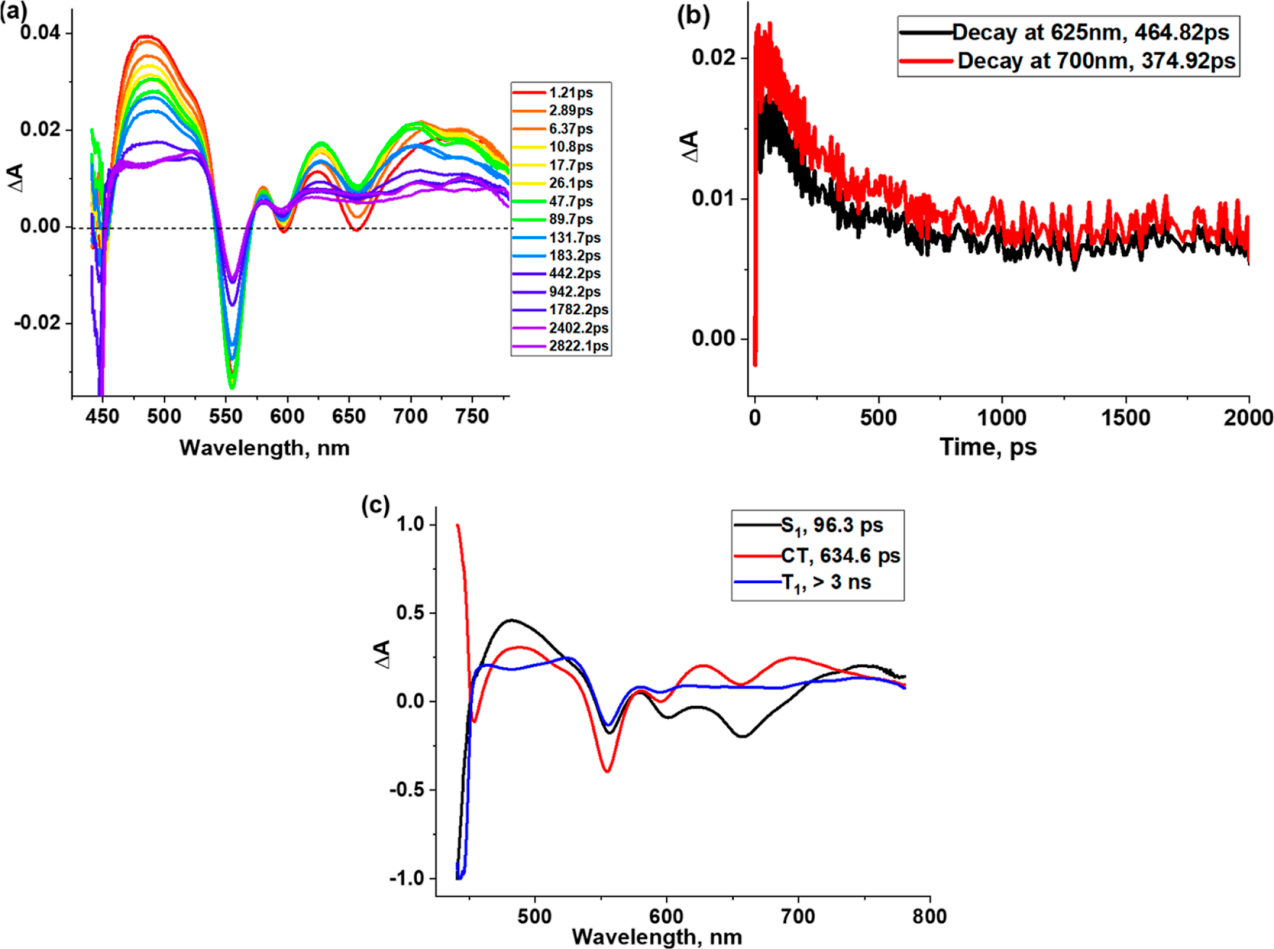
(a) *fs*-TA spectra at the indicated delay times of SbT(DMP)P(OTFE)_2_·PF_6_ in CH_2_Cl_2_ with an excitation wavelength of 435 nm. (b) Time profile of the 625 and 700 nm peaks. (c) Decay-associated spectra from glotaran analysis.

On the contrary, the fs-TA spectra of SbT(DMP)P(OTFE)_2_·PF_6_ in CH_2_Cl_2_ at the excitation wavelength of 435 nm, as shown in Figure 9a, revealed the intermediate formation of charge transfer. In this instance, the initially formed S_1_ state revealed ESA peaks at 485, 579, 626, and 720 nm and GSB/SE peaks at 555, 597, and 655 nm. The decay and recovery of the positive and negative peaks were accompanied by a new peak at 700 nm, expected for the charge-transfer state (see the spectrum at 47.7 ps in Figure 9a). With time, decay/recovery of the spectral features was associated with new peaks at 467, 523, 722, and 762 nm attributable to the T_1_ state (see spectrum at 2822 ps). The time profiles of the 625 and 700 nm peaks, attributable to the charge-transfer state, are shown in Figure 9b. Decay time constants of 465 and 375 ps were observed. Further, the data was subjected to glotaran analysis by fitting the data to the S_1_ → CT → T_1_ model (Figure 9c). From global analysis lifetimes of 634 ps for the CT state and >3 ns for the T_1_ state were possible to arrive.

**Figure 10:**
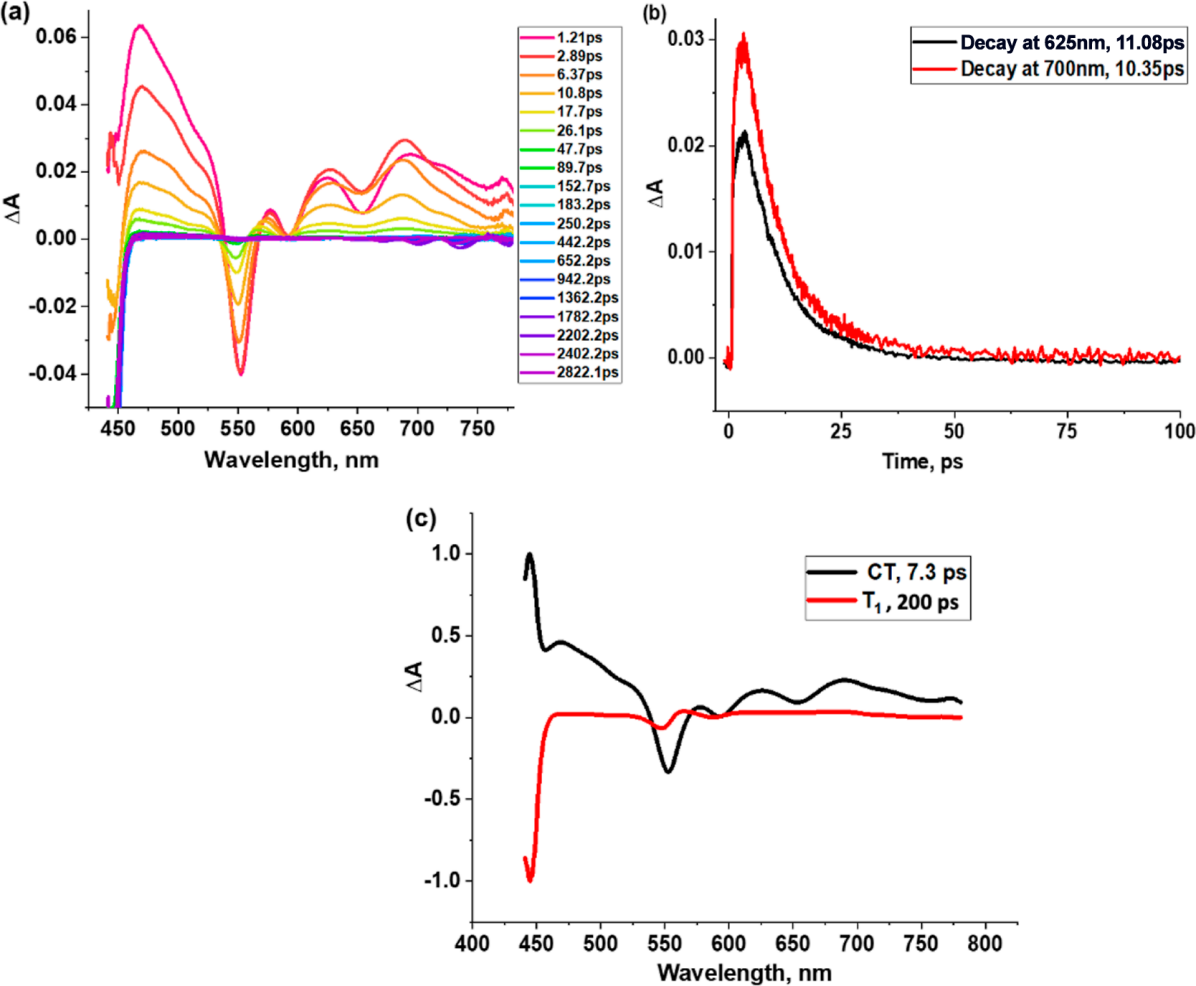
(a) *fs*-TA spectra at the indicated delay times of SbT(DMP)P(OTFE)_2_·PF_6_ in CH_3_CN with an excitation wavelength of 435 nm. (b) Time profile of the 625 and 700 nm peaks. (c) Decay-associated spectra from glotaran analysis.

Finally, *fs*-TA spectra data was secured in polar CH_3_CN, as shown in Figure 10a. The spectral features at the earliest recordable delay time closely matched those of the charge-transfer state. That is, the S_1_ state formed within the subpicosecond delay time rapidly transformed into the CT state. ESA peaks at 468, 577, 624, and 693 nm and GSB and SE peaks at 552, 591, and 654 nm. The decay of the charge-transfer peaks showed weak features of the triplet state at longer delay times. Decay time profiles of the 625 and 700 nm peaks, corresponding to the charge-transfer state, are shown in Figure 10b. Decay time constants of 11.1 and 10.4 ps were obtained. glotaran analysis of the transient data shown in Figure 10c revealed the CT spectrum with a time constant of 7.3 ps and a weak triplet state with a lifetime of around 200 ps.

## Discussion

The study shows the synthesis of antimony(V) porphyrins showcasing efficient ICT in a fairly straightforward manner relative to the many complex molecular systems found in literature.^35−38^ The high oxidation state of the antimony(+5) ion is a crucial factor to induce the ICT in the molecule. The study further shows that the charge-transfer phenomenon is not only induced but also can be fully controlled structurally. The Soret regions of the absorption spectra of SbT(DMP)P(OMe)_2_·PF_6_ and SbT(DMP)P(OTFE)_2_·PF_6_ are significantly broader than that of the reference compound, SbTPP(OMe)_2_·PF_6_. The broadness increases with an increase in the electron disparity between the central and peripherals of the molecules. Such electron disparity is also manifested in solvatochromism, where SbT(DMP)P(OTFE)_2_·PF_6_ is more sensitive to solvent polarity than SbT(DMP)P(OMe)_2_·PF_6_. The SbT(DMP)P(OTFE)_2_·PF_6_ becomes nonemissive in moderate to polar solvents, whereas SbT(DMP)P(OMe)_2_·PF_6_ is nonemissive in only highly polar solvents. The emissive response as a function of polarity is direct evidence for the presence of ICT in the studied molecules. This evidence is further complimented by steady-state fluorescence studies. Hence, all the steady-state studies indicate that both SbT(DMP)P(OMe)_2_·PF_6_ and SbT(DMP)P(OTFE)_2_·PF_6_ possess an ICT character and suggest that SbT(DMP)P(OTFE)_2_·PF_6_ has stronger ICT than SbT(DMP)P(OMe)_2_·PF_6_.

Electrochemical studies revealed two reductions and one oxidation process for each of the studied systems. Interestingly, the first reduction potential of SbT(DMP)P(OTFE)_2_·PF_6_ is only 20 mV lower than that of SbT(DMP)P(OMe)_2_·PF_6_. On the other hand, the oxidation potentials went up slightly by 10 mV for SbT(DMP)P(OTFE)_2_·PF_6_ when compared to the SbT(DMP)P(OMe)_2_·PF_6_. Overall, these potentials resulted in very similar energies for the charge-separated states in their energy diagrams, that is, 1.82 eV for SbT(DMP)P(OMe)_2_·PF_6_ and 1.80 eV for SbT(DMP)P(OTFE)_2_·PF_6_. These energies show that the driving force is slightly more in SbT(DMP)P(OTFE)_2_·PF_6_. Despite the very similar redox potentials and excited state energies, their optical properties are significantly different as the ICT character is more strongly pronounced in SbT(DMP)P(OTFE)_2_·PF_6_ than in SbT(DMP)P(OMe)_2_·PF_6_.

Finally, it was possible to spectrally characterize the charge-transfer species in SbT(DMP)P(OTFE)_2_·PF_6_ and secure the kinetic information by using pump–probe studies. The observed ICT lifetime of 7.3 ps for SbT(DMP)P(OTFE)_2_·PF_6_ is shorter than that of 20.8 ps for SbT(DMP)P(OMe)_2_·PF_6_ in CH_3_CN, further suggesting that the ICT is more pronounced in SbT(DMP)P(OTFE)_2_·PF_6_ which compliments the solvatochromism studies. Moreover, the pump–probe studies of SbT(DMP)P(OTFE)_2_·PF_6_ indicate that the porphyrin excited state decays either to the ground state or to the CT state followed by to the triplet state depending on the solvent polarity. In nonpolar toluene, the excited state simply decays to the ground state via a fluorescence process. In moderately polar CH_2_Cl_2_ and strongly polar CH_3_CN, the resulting CT state decays to the triplet state through intersystem crossing; the resulting triplet state survives >3 ns and 200 ps, respectively. The lifetime of the final charge-transfer states was ten to hundreds of picoseconds depending upon the solvent revealing them to be promising for building the next generation of energy harvesting devices. Further studies along this line are in progress in our laboratories.

## Conclusions

In summary, control over charge transfer in antimony(V) porphyrins by the choice of axial ligand (electron-rich or -deficient) and peripheral electron-rich substituents has been demonstrated. From a combination of absorption, steady-state and time-resolved emission, electrochemistry, and computational studies, it was possible to demonstrate the role of the electron-deficient fluoro-alkyl entity in promoting charge transfer not only in polar CH_3_CN but also in a moderately polar CH_2_Cl_2_, for an antimony(V) porphyrin carrying the same electron-rich peripheral substituents. This was unlike the case of simple antimony(V) porphyrin carrying methoxy substituents, where charge transfer was possible to witness only in polar CH_3_CN and not in medium polar and nonpolar solvents. The present study brings out the importance of the electron donating/withdrawing nature of axial ligands in governing ICT.

## Supporting Information Available

ESI mass spectrum, NMR spectra, absorption and fluorescence spectra, fluorescence decay profiles, spectroelectrochemistry data, and transient absorption spectra (PDF)
