# Supplementary material for: Tuning Intramolecular Charge Transfer in Antimony(V) Porphyrin through Axial Fluorination
Source: ACS Omega. 2024 May 13;9(21):22892–902. doi: 10.1021/acsomega.4c01773 (PMC11137685; doi:10.1021/acsomega.4c01773)
Supplement: Supplementary file 1 — ao4c01773_si_001.pdf [file ao4c01773_si_001.pdf]

Supporting Information

Tuning Intramolecular Charge Transfer in Antimony(V) Porphyrin through Axial Fluorination

Noah Holzer,[a,b,#] *Jatan K. Sharma,*[c,#] *Francis D'Souza,*[c,*] *Prashanth K. Poddutoori*[a,b,*]

[a]*Advanced Materials Center, University of Minnesota Duluth, 1405 University Drive, Duluth, Minnesota 55812, USA.*

[b]*Department of Chemistry & Biochemistry, University of Minnesota Duluth, 1038 University Drive, Duluth, Minnesota 55812, USA.*

[c]*Department of Chemistry, University of North Texas, 1155 Union Circle, # 305070, Denton, Texas 76203-5017, USA.*

[#]Equal contribution

Physical methods

NMR and mass spectroscopy. NMR spectra were recorded on a Bruker Advance 400 MHz NMR spectrometer using $CDCl_3$ as the solvent. ESI mass spectra were recorded on a Bruker MicroTOF-III mass spectrometer using direct injection from an UltiMate 3000 HPLC and CH_3CN as a solvent.

Absorption and emission spectroscopy. UV/Vis spectra were recorded with an Agilent Cary 100 UV/Vis spectrometer. The concentration of the samples used for these measurements ranged from 5×10^{-6} M (porphyrin B- band (Soret)) to 5×10^{-5} M (Q-bands) solutions. Steady-state fluorescence spectra were recorded using a Photon Technologies International Quanta Master 8075-11 spectrofluorometer, equipped with a 75 W Xenon lamp, running with FelixGX software. An excitation wavelength of 550 nm was used, and the optical density was held constant at 0.2 for all of the compounds.

Electrochemistry. Cyclic voltammetric experiments (CH_3CN 0.1 M tetrabutylammonium hexafluorophosphate ($TBA\cdot PF_6$) were performed on a Parstat 4000A electrochemical analyzer (Pt working electrode; Pt wire auxiliary electrode; Ag wire reference electrode). The Fc^+/Fc (Fc = ferrocene, $E_{1/2}(Fc^+/Fc)$ = 0.40 V *vs* SCE in CH_3CN, 0.1 M $TBA\cdot PF_6$ under our experimental conditions)[1] redox couple was used to calibrate the potentials.

DFT calculations. DFT computations were completed using *Gaussian 16*.[2] Initial geometries were created based on the crystal structure of $SbT(DMP)P(OMe)_2 \cdot PF_6$. The B3LYP functional was used with multiple basis sets via the GenECP keyword. The 6-311G(d,p) basis set was used to model the light elements (H, C, N, O, F) whereas the antimony ion was modeled instead using the def2TZVPP basis set along with its integrated effective core potentials. The structures were optimized in vacuum as closed-shell singlet mono-cations without the use of symmetry constraints. The self-consistent field convergence criterion was modified to 10^{-8}; requiring that the RMS change in the density matrix converge to $< 10^{-8}$ and that the maximum change in the density matrix be $< 10^{-6}$. The convergence criteria for the DFT grid and the geometrical parameters were kept at the *Gaussian 16* defaults.

Time-resolved fluorescence spectroscopy. A time-correlated single-photon-counting apparatus utilizing a picosecond-pulsed diode laser was used to measure the porphyrin fluorescence decay. Excitation pulses were delivered at ~560 nm by a picosecond diode laser (PicoQuant, PDL 800-B), 54 ps FWHM, at a repetition rate of 10 MHz. The porphyrin fluorescence was measured by a Hamamatsu R3809 microchannel plate photomultiplier screened by a double monochromator. A single-photon-counting PC card (Becker & Hickl, SPC-730) was used for data collection. The instrument response time of the system was 80 ps.

Femtosecond laser flash photolysis. Femtosecond transient absorption spectroscopy. These experiments were performed using an ultrafast femtosecond laser source (Libra) by Coherent incorporating a diode-pumped, mode-locked Ti: sapphire laser (Vitesse) and a diode-pumped intracavity doubled Nd: YLF laser (Evolution) to generate a compressed laser output of 1.45 W. For optical detection, a Helios transient absorption spectrometer coupled with a femtosecond harmonics generator, both provided by Ultrafast Systems LLC, was used. The sources for the pump and probe pulses were derived from the fundamental output of Libra (Compressed output 1.45 W, pulse width 100 fs) at a repetition rate of 1 kHz; 95% of the fundamental output of the laser was introduced into a TOPAS-Prime-OPA system with a 290−2600 nm tuning range from Altos Photonics Inc., (Bozeman, MT), while the rest of the output was used for generation of a white light continuum. Kinetic traces at appropriate wavelengths were assembled from the time-resolved spectral data. Data analysis was performed using Surface Xplorer software supplied by Ultrafast Systems. All measurements were conducted in degassed solutions at 298 K. The estimated error in the reported rate constants is ±10%.

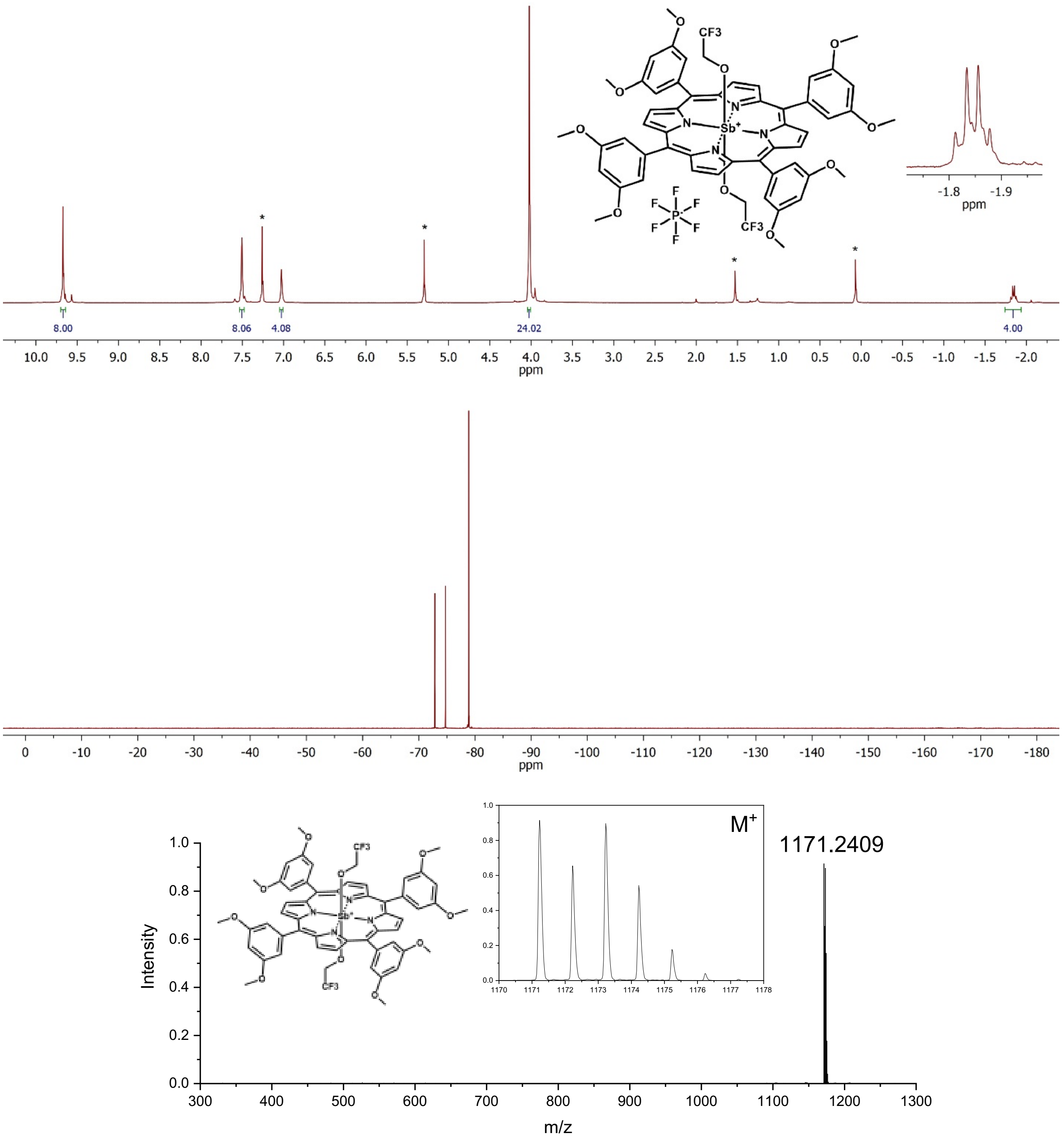

Figure S1. ^{1}H, ^{19}F, and ESI-mass spectra of the newly synthesized SbT(DMP)P(OTFE)$_2$·PF$_6$.

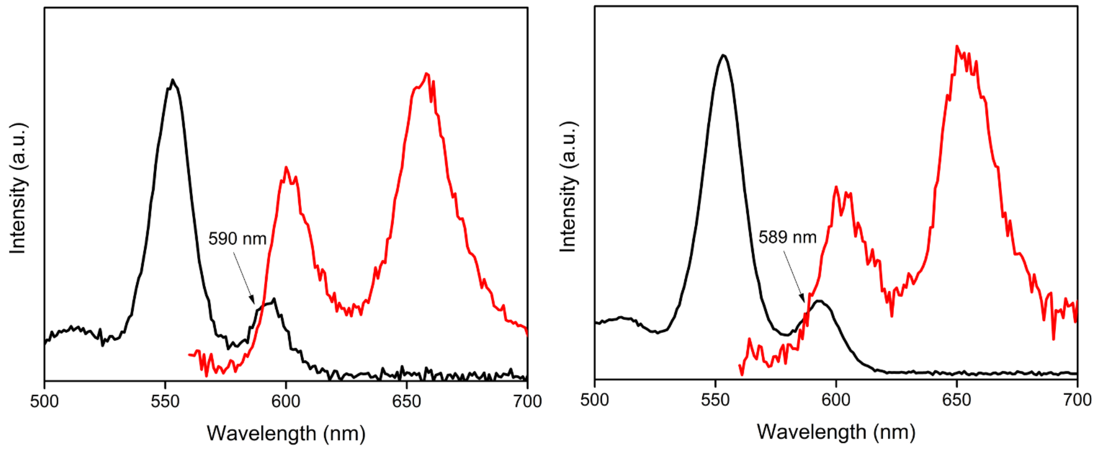

Figure S2. Absorption (black) and fluorescence (red) spectral overlap of SbT(DMP)P(OMe)$_2$·PF$_6$ and SbT(DMP)P(OTFE)$_2$·PF$_6$ in CH_3CN.

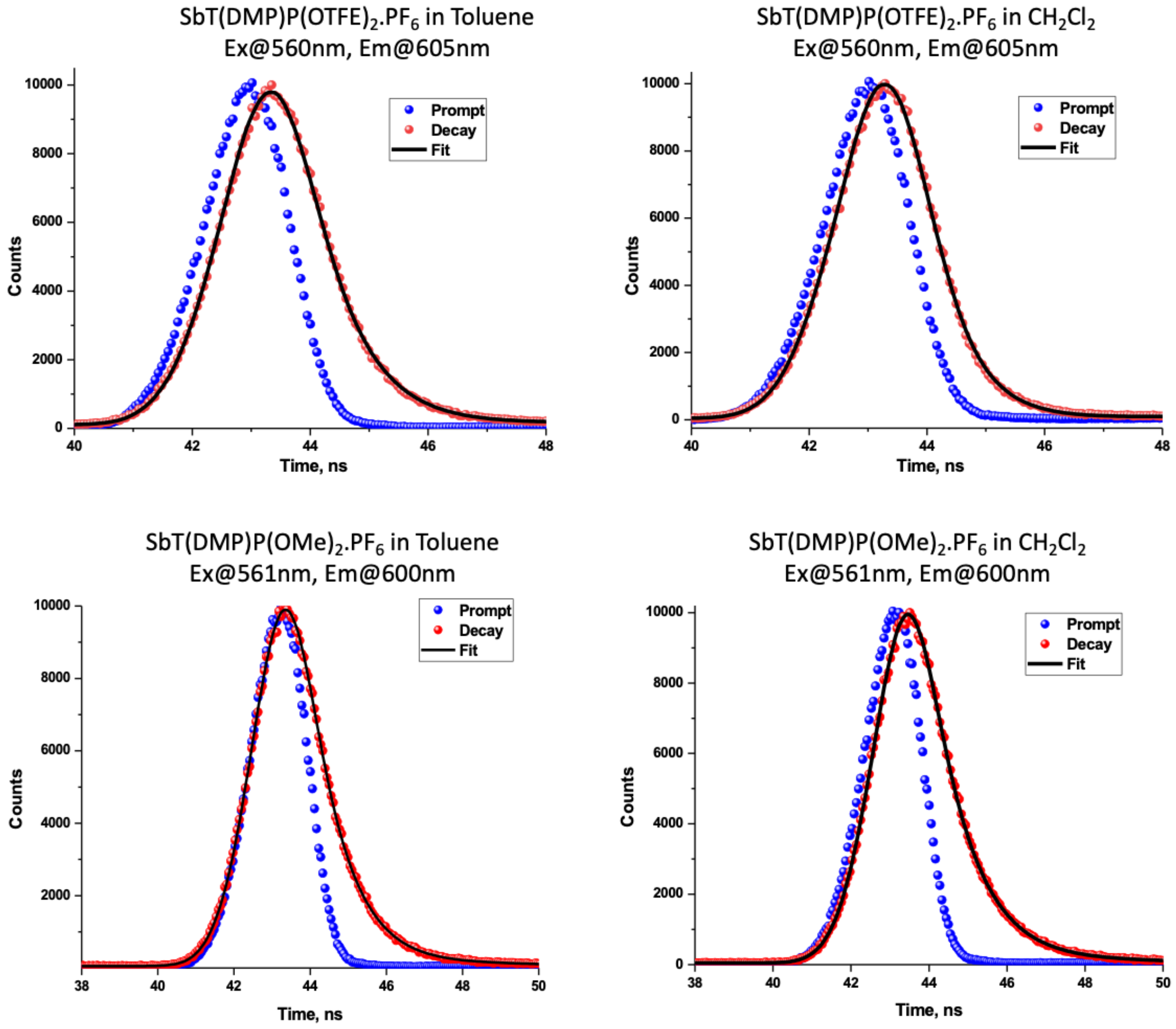

Figure S3. Fluorescence decay profiles of SbT(DMP)P(OTFE)$_2$·PF$_6$ and SbDMP(OMe)$_2$·PF$_6$ in toluene and CH_2Cl_2.

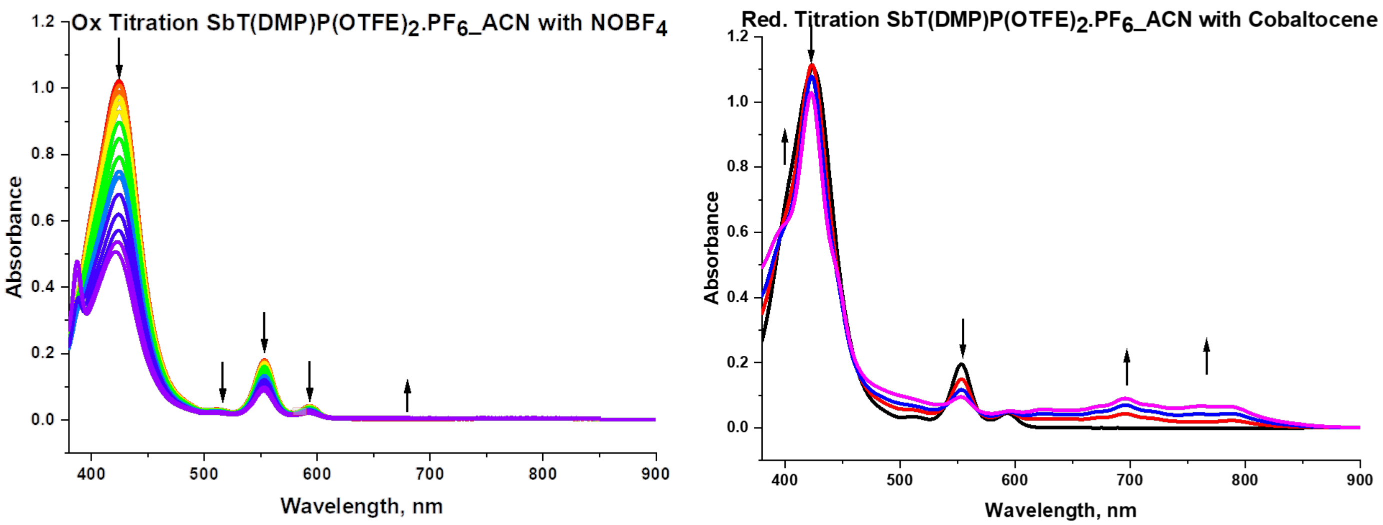

Figure S4. Spectral changes during chemical oxidation and chemical reduction of SbT(DMP)P(OTFE)$_2$·PF$_6$ in CH$_3$CN. Nitrosonium tetrafluoroborate as an oxidizing agent and cobaltocene as the reducing agent was utilized.

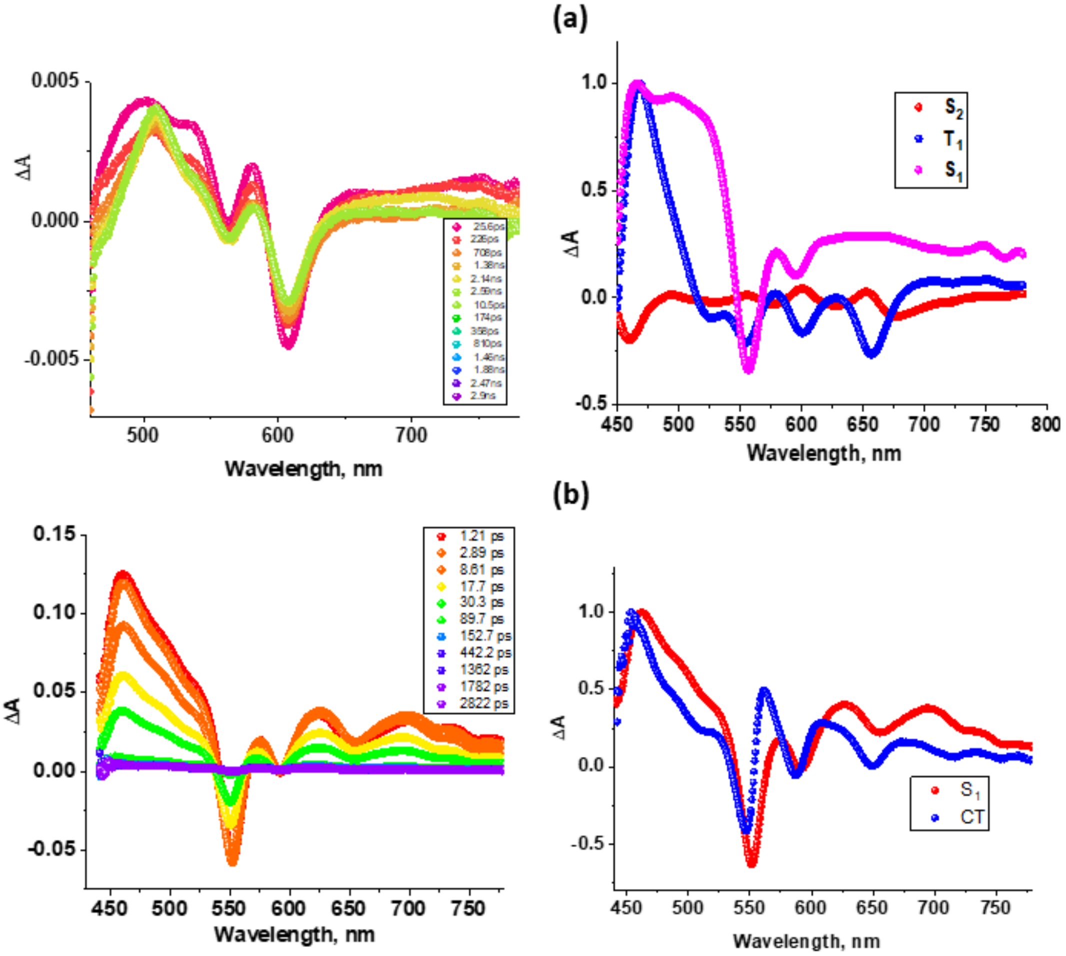

Figure S5. *fs*-TA absorption spectra at the indicated delay times of SbT(DMP)P(OMe)$_2$.PF$_6$ in oxygen-free (a) toluene and (b) acetonitrile at the Soret band excitation. The corresponding decay associated generated from the global analysis is shown on the right-hand side. Reproduced from Ref. 3 with permission from the Royal Society of Chemistry.

References

(1) Connelly, N. G.; Geiger, W. E. Chemical Redox Agents for Organometallic Chemistry. *Chem Rev* **1996**, *96* (2), 877–910. https://doi.org/10.1021/cr940053x.

(2) Frisch, M. J.; Trucks, G. W.; Schlegel, H. B.; Scuseria, G. E.; Robb, M. a.; Cheeseman, J. R.; Montgomery, J. a.; Vreven, T.; Kudin, K. N.; Burant, J. C.; Millam, J. M.; Iyengar, S. S.; Tomasi, J.; Barone, V.; Mennucci, B.; Cossi, M.; Scalmani, G.; Rega, N.; Petersson, G. a.; Nakatsuji, H.; Hada, M.; Ehara, M.; Toyota, K.; Fukuda, R.; Hasegawa, J.; Ishida, H.; Nakajima, T.; Honda, Y.; Kitao, O.; Nakai, H.; Klene, M.; Li, X.; Knox, J. E.; Hratchian, H. P.; Cross, J. B.; Adamo, C.; Jaramillo, J.; Gomperts, R.; Stratmann, R. E.; Yazyev, O.; Austin, A. J.; Cammi, R.; Pomelli, C.; Ochterski, J.; Ayala, P. Y.; Morokuma, K.; Voth, G. a.; Salvador, P.; Dannenberg, J. J.; Zakrzewski, V. G.; Dapprich, S.; Daniels, A. D.; Strain, M. C.; Farkas, O.; Malick, D. K.; Rabuck, A. D.; Raghavachari, K.; Foresman, J. B.; Ortiz, J. V.; Cui, Q.; Baboul, A. G.; Clifford, S.; Cioslowski, J.; Stefanov, B. B.; Liu, G.; Liashenko, A.; Piskorz, P.; Komaromi, I.; Martin, R. L.; Fox, D. J.; Keith, T.; Al-Laham, M. a.; Peng, C. Y.; Nanayakkara, A.; Challacombe, M.; Gill, P. M. W.; Johnson, B.; Chen, W.; Wong, M. W.; Gonzalez, C.; Pople, J. a. Gaussian 16. Gaussian Inc.: Wallingford, CT 2016.

(3) Holzer, N.; Sharma, J. K.; Peterson, S.; Bayard, B. J.; Nesterov, V. N.; Karr, P. A.; D'Souza, F.; Poddutoori, P. K. Antimony(+5) Ion Induced Tunable Intramolecular Charge Transfer in Hypervalent Antimony(v) Porphyrins. *Dalton Transactions* **2022**, *51* (15), 5890–5903. https://doi.org/10.1039/d2dt00675h.
